# Exercise responsive genes measured in peripheral blood of women with Chronic Fatigue Syndrome and matched control subjects

**DOI:** 10.1186/1472-6793-5-5

**Published:** 2005-03-24

**Authors:** Toni Whistler, James F Jones, Elizabeth R Unger, Suzanne D Vernon

**Affiliations:** 1 Viral Exanthems and Herpesvirus Branch, Centers for Disease Control and Prevention, Atlanta, GA 30333, USA

## Abstract

**Background:**

Chronic fatigue syndrome (CFS) is defined by debilitating fatigue that is exacerbated by physical or mental exertion. To search for markers of CFS-associated post-exertional fatigue, we measured peripheral blood gene expression profiles of women with CFS and matched controls before and after exercise challenge.

**Results:**

Women with CFS and healthy, age-matched, sedentary controls were exercised on a stationary bicycle at 70% of their predicted maximum workload. Blood was obtained before and after the challenge, total RNA was extracted from mononuclear cells, and signal intensity of the labeled cDNA hybridized to a 3800-gene oligonucleotide microarray was measured. We identified differences in gene expression among and between subject groups before and after exercise challenge and evaluated differences in terms of Gene Ontology categories.

Exercise-responsive genes differed between CFS patients and controls. These were in genes classified in chromatin and nucleosome assembly, cytoplasmic vesicles, membrane transport, and G protein-coupled receptor ontologies. Differences in ion transport and ion channel activity were evident at baseline and were exaggerated after exercise, as evidenced by greater numbers of differentially expressed genes in these molecular functions.

**Conclusion:**

These results highlight the potential use of an exercise challenge combined with microarray gene expression analysis in identifying gene ontologies associated with CFS.

## Background

In a state of health, physical exercise has a quantifiable effect on neuroendocrine, autonomic, and immune systems influencing metabolic and immune responses. However, in the initial phase of acute illness, there is an avoidance of physical stressors so energy can be dedicated to healing and a return to homeostasis. While physiologic disturbance in acute illness is transient, chronic illnesses, such as chronic fatigue syndrome (CFS), have prolonged disturbances that have a debilitating effect both physiologically and psychologically. Consequently, activities that are physiologic stressors, such as physical exercise, exacerbate the symptoms that define CFS.

CFS is a complex, multifactorial illness whose etiology and pathophysiology remain unclear [1]. CFS is defined by a characteristic symptom complex in the absence of other medical or psychiatric conditions with similar clinical characteristics [2,3]. Subtle differences in hypothalamic-pituitary-adrenal axis function [4], immune system function [5], and psychological profiles [6] between CFS patients and controls have been reported; however, no consistent distinguishing difference or frank abnormality has been confirmed [7,8], and it remains unclear whether CFS represents a unique disease or a common illness response to a variety of insults.

Perhaps the greatest methodological problem with studying CFS is that many individuals identified in population studies have been sick for at least 5 years [9]. During this time, the illness waxes and wanes, making it difficult to identify biomarkers or define pathogenesis. Physical, mental, and emotional stress exacerbate CFS and result in case-defining post-exertional fatigue [2] with measurable physiologic differences [10]. Therefore, exercise challenge of people with CFS is an effective method for calibrating CFS subjects and thus increasing the likelihood of uniformly identifying biomarkers and/or physiologic abnormalities.

We used gene expression profiling of peripheral blood to evaluate differences between CFS subjects and sedentary healthy controls both before and following an exercise challenge. Overall, we found the gene expression profiles to be quite similar, and of importance, most differences were present prior to exercise challenge. These differences were in G protein-coupled receptor and ion transport and ion channel activity ontologies. The latter was exaggerated after exercise as evidenced by differential expression of a greater number of genes involved in these molecular functions. Differences were also evident in exercise response, including chromatin and nucleosome assembly, cytoplasmic vesicles, membrane transport and G-protein coupled receptor ontologies. These differences may help explain the symptoms of CFS.

## Results

Exercise response genes were evaluated using a random variance t test in a paired, class comparison analysis of control subjects before and after exercise, and 21 genes were identified as being differentially expressed (Table 2). The probability of identifying these 21 genes by chance if there are no real differences between the classes was 0.056 as determined by the multivariate permutation test. Among the 21 genes, 16 could be categorized in the Gene Ontology (GO) of biological process and 15 in molecular function (results not shown). The most significant categories or "themes" of these exercise-responsive genes as assessed by an EASE score of <0.10, pertained to the biological process of transport (both vesicle-mediated and protein transport). 5 of the 21 genes were involved in this process.

**Figure 1:**
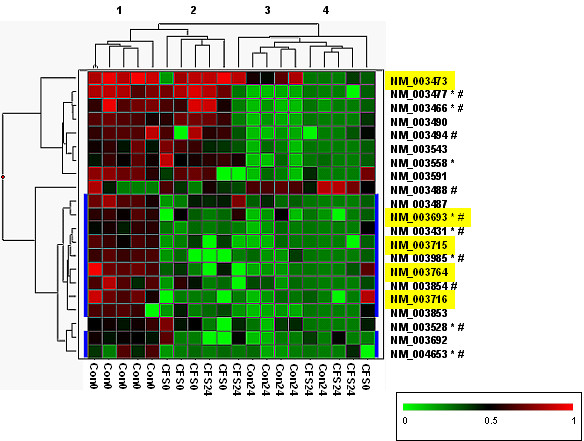
**Hierarchical clustering of exercise responsive genes in control subjects. **The 21 differentially expressed genes identified by a class comparison test of control subjects (before and after exercise challenge (Table 2)) were clustered using a two-way hierarchical algorithm. In the matrix each row represents the hybridization results for a single gene, and each column represents a subject. Transcript levels are depicted as above (red) or below (green) the mean. The dendograms illustrates average-linkage hierarchical clustering of subjects (top) and genes (left). Refseq IDs for each gene is given on the right of the matrix. Those with similar exercise responses in both CFS and control subjects are at the top of the matrix, and the remainder of genes (highlighted in blue) show a diminished exercise response in CFS cases. Refseq IDs highlighted in yellow classify to the GO categories of protein or vesicle-mediated transport (Biological Process). Refseq IDs followed by: 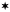 are classified to the GO category of binding (Molecular Function); and/or # are classified in the GO category of metabolism (Biological Process). The subjects group into 4 clusters which approximate to: 1) Control subjects before exercise (Con0); 2) CFS cases before exercise (CFS0); 3) Control subjects after exercise (Con24); and 4) CFS cases after exercise (CFS24).

Since these 21 genes reflect a healthy subject's peripheral blood gene expression response to exercise challenge, we reasoned that the expression of these would be altered in CFS subjects. To have a visual representation of these differences, the gene list from Table 2 (differentially expressed genes in control subjects, compared before and after exercise challenge) was used in a two-way hierarchical cluster analysis (Figure 1). The response of 10 of the 21 genes was quite similar in terms of magnitude and direction for both CFS and control subjects (Figure 1, marked in blue). For the other 11 genes, the magnitude of the exercise change was considerably smaller in CFS subjects (Figure 1, subject clusters 2 and 4) than in control subjects (clusters 1 and 3). With regard to the GO categories of these 21 genes, 10 genes were associated with binding and 8 with metabolism, all of which were equally distributed between the two response types (denoted by # and 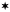 in Figure 1). However, 5 genes classified in vesicle-mediated and protein-transport ontologies differed between CFS and control subjects (Figure 1, highlighted in yellow).

No differentially expressed genes were identified by class comparison analysis (at a significance level of p > 0.005) for CFS subjects before and after exercise, for CFS subjects before exercise compared with controls before exercise, or for CFS subjects after exercise compared with controls after exercise.

**Figure 2:**
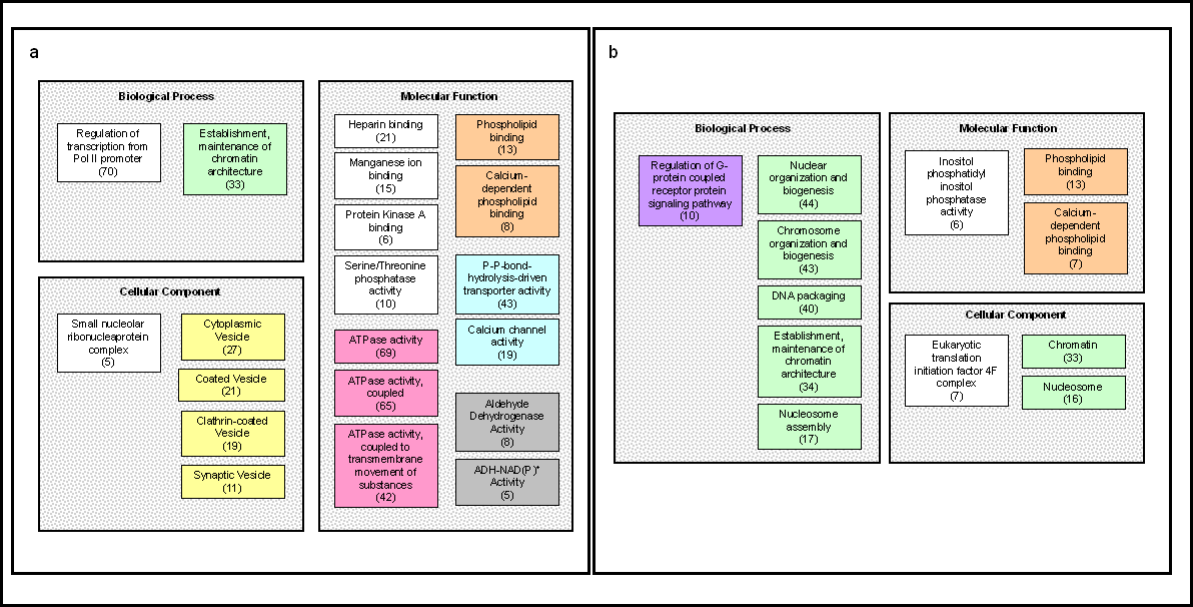
**Significant gene ontology categories defining exercise-related changes in control (a) and CFS subjects (b). **The three organizing principles of GO (represented as grey shaded boxes) are molecular function, biological process, and cellular component. Related ontologies and/or subgroups of the ontologies are denoted by similarly colored squares in all tables. Ontologies presented in these figures were significant at a p-value <0.005 by one or both of the LS and KS permutation tests.

**Figure 3:**
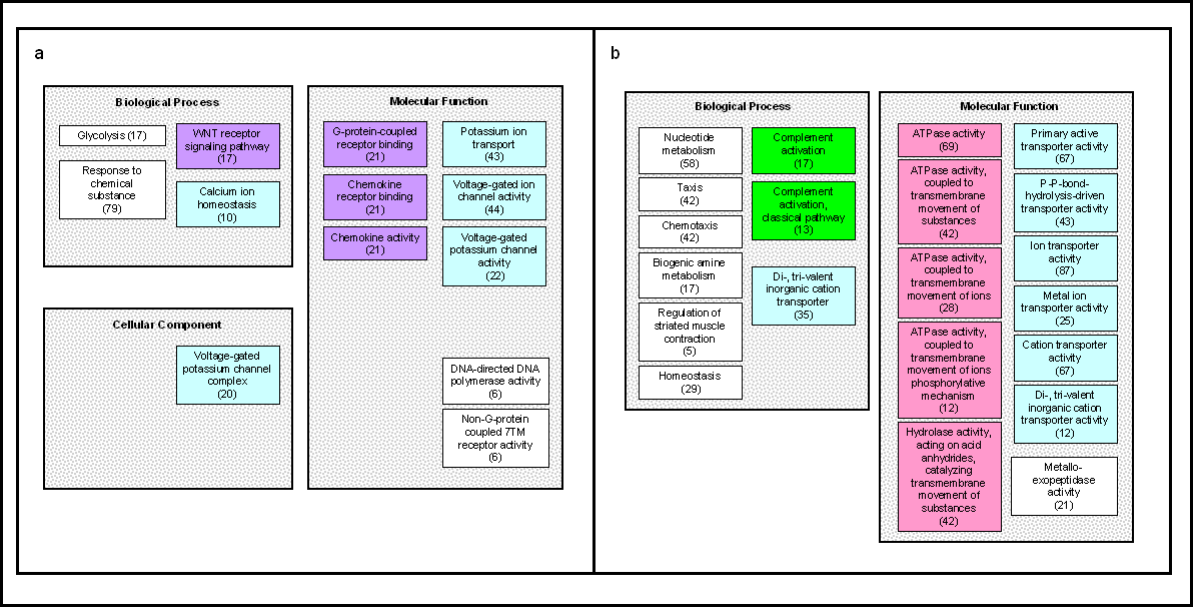
**Gene Ontology categories identified as significantly different between controls and CFS cases at baseline (pre-exercise) (a) and post-exercise (b). **The three organizing principles of GO (represented by grey shaded squares) are molecular function, biological process, and cellular component. Related ontologies and/or subgroups of the ontologies are denoted by similarly colored squares in all tables. Ontologies presented in these figures were significant at a p-value <0.005 by one or both of the LS and KS permutation tests.

Because differentially expressed genes were identified by class comparison prior to the exercise challenge, we reasoned that a comparison of gene expression in CFS and control subjects, using genes categorized by ontology, would more efficiently reveal perturbed physiological pathways. Figures 2 and 3 show the results of these analyses. GO terms with defined parent-child relationships are grouped together in the figures and are color-matched. Only two exercise-responsive GO categories [phospholipid binding (orange) and chromatin architecture (pale green)] were common to controls and CFS subjects (Figure 2a and 2b). However, for the chromatin architecture category, the CFS comparison highlighted 7 overlapping ontologies (containing 59 unique genes), compared with 1 ontology of 33 genes in the control comparison. The 33 genes overlap with the 59 identified in the CFS comparison. The phospholipids-binding ontologies were identical in gene composition. Exercise-related changes that were identified as significant only in control subjects were associated with genes involved with vesicle (yellow, Figure 2a), dehydrogenase (grey, Figure 2a), ATPase (pink, Figure 2a), and transporter (blue, Figure 2a), activities. Exercise-related changes that were seen only in CFS subjects were related to G-protein-coupled receptor signaling (purple, Figure 2b).

Gene ontology comparison was also used to evaluate differences between control and CFS subjects before (i.e. baseline, Figure 3a) and after (Figure 3b) exercise. Baseline differences between CFS subjects and controls that continued after exercise involved GO terms relating to ion transport (blue, Figure 3a). After exercise, these differences appear to be amplified, as evidenced by increased numbers of genes present in these GO categories and also by inclusion of more GO terms pertaining to ATPase transmembrane movement of ions (pink, Figure 3b). G-protein-coupled receptor binding (purple, Figure 3a), part of the broad functional category of signal transduction, differed between CFS subjects and controls prior to exercise. This baseline difference between controls and CFS subjects was not significant after exercise. Interestingly, complement activation (dark green, Figure 3b) was one of the exercise-induced differences between subjects and controls that was present only after challenge. Genes in most of the ontologies identified as different between CFS and control subjects had lower expression levels in CFS subjects.

## Discussion

Gene expression profiling affords a unique opportunity to characterize CFS at a systems biology level. Changes in gene expression underlie many biologic processes and may provide insight into disease-specific gene expression and the response of genes to environmental stimuli. In a proof-of-concept study, we found that CFS patients had different blood mononuclear cell gene expression patterns than non-fatigued controls [11] and that CFS is a heterogeneous illness as evidenced by different gene expression profiles for patients reporting gradual onset of their illness compared with those reporting sudden onset of illness [12]. In addition, differential display polymerase chain reaction on a small number of CFS and control subjects identified candidate biomarkers in the peripheral blood [13,14].

CFS is defined by a post-exertional fatigue that does not subside 24 hours following physical stress. In contrast, exercise in healthy, untrained people induces changes in cellular homeostasis in 1 to 4 hours and a return to basal levels within 24 hours, as measured in muscle [15]. Analysis of peripheral blood gene expression in the healthy control subjects confirmed this observation since the majority of gene expression levels were the same before and 24 hours following exercise challenge. This implied that expression either returned to basal levels or was unchanged as a result of the exercise challenge. And indeed, many of the 21 exercise-induced, differentially expressed genes in control subjects were characterized by GOs that reflect a diverse set of molecular functions necessary for cell function and viability. (These ontologies overlapped with those identified in the GO comparison analysis given in Figure 2a). Figure 1 clearly illustrates the reciprocal pattern of gene expression in the 21 genes for most of the control subjects. In contrast, 11 of the genes were unchanged in CFS subjects before and after exercise; with 5 being classified in a transport-related ontology. Because this difference in gene expression is so dramatic, it implicates a fundamental perturbation in the biochemical activity of lymphocyte and monocyte peripheral blood fractions from CFS subjects compared with control subjects that does not affect classical immunologic markers (i.e, CD45) that have been shown to be unaffected in CFS patients [16,17]. Rather, low expression of these genes may have subtle effects on immune function. Immune dysfunction has been inconsistently implicated in CFS pathogenesis [18].

Class comparison was used to identify these 21 differentially expressed genes, which indicated the possible disturbance of biologic pathways (Figure 1). To explore this possibility, we used the GO comparison that is based on the knowledge that gene expression levels are dependent variables in biological processes, cellular components, and molecular functions. In this way, multiple genes in the same category reinforce each other and enhance the power for identifying the significance of the category. The GO categories considered significantly different (p < 0.005) when comparing CFS subjects with controls after exercise challenge were those pertaining to ion transporter activity (a total of 87 genes applied to this category in the comparison of CFS and controls after exercise) and ATPase activity coupled to transmembrane movement (42 genes). When the CFS and control classes are compared prior to exercise, ion transport activity and voltage-gated, ion channel activity are identified (38 and 44 genes within the GO categories, respectively).

It is evident that ion transport and ion channel activity segregate cases from controls and that exercise seems to intensify these differences. Several other conditions have been reported in which fluctuating fatigue occurs that are known to be caused by abnormal ion channels. These conditions include genetically determined channelopathies and acquired conditions such as neuromyotonia, myasthenic syndromes, multiple sclerosis, and polyneuropathies [19,20]. There are other transmembrane functions associated with differences between controls and CFS patients, including signal transducer activity through receptor binding/activity (Figure 3a). Signal transduction of transmembrane receptors occurs by a number of mechanisms, including structural changes, ion channels, and changes of transmembrane potentials. The G-protein-coupled receptors play an important role in the membrane trafficking machinery [21]. The most obvious exercise-induced changes in CFS cases pertain to gene regulation at the point of chromatin structure; whether these changes reflect the differences seen in the mRNA transcripts relating to membrane trafficking differences between cases and controls has not yet been determined.

One interesting correlate of this study was the finding that the complement pathway showed significant differences between CFS and control subjects after exercise. This has been reported previously in the analysis of these same exercise challenge-derived specimens. Sorensen et al. [22] measured levels of complement split products in the sera of these subjects and found differences between CFS and control subjects in C4a after exercise challenge. Complement activation was identified as an ontology that was significantly different between CFS and control subjects after exercise. The correlates on the data are interesting as their study measured protein levels (i.e., gene product levels) and this study measured the transcript levels.

The class comparison analysis performed in this study accounted for multiple testing and the over fitting problems of microarray data analysis. The lack of statistical significance in the 3 other class comparison analyses performed (CFS cases compared before and after exercise, comparison of cases to controls at baseline, and the comparison of cases to controls 24 hours after exercise) reflects low experimental sensitivity, most likely due to a small number of subjects, rather than an absence of biological effect. This is accounted for in the gene ontology comparison tool where classes are compared by GO category rather than with regard to individual genes.

The next line of research will detail larger numbers of subjects in the expression arrays. The emphasis in such studies will be on developing a gene expression-based multivariate function, or predictor, that accurately predicts the class membership of a new sample on the basis of the expression levels of key genes. Class discovery tools will also be applied to CFS subjects' expression profiles in an attempt to further describe discrete subsets of this disease on the basis of gene expression as we have done for gradual and sudden onset of illness [12]. However, the methods used in this study will be applied to these data sets too, as these analytical tools will prove to be very helpful in defining the pathophysiology of CFS. It is hoped that this broader, more fully encompassing approach to CFS research will open many doors to the understanding of this syndrome and perhaps of fatigue and un-wellness in general.

## Methods

### Study subjects

This study adhered to human experimentation guidelines of the U.S. Department of Health and Human Services and the Helsinki Declaration. All participants were volunteers who gave informed consent. Study parameters have been described [22]. In brief, women who attended a CFS outpatient fatigue clinic volunteered for the study. All of these patients met the current working definition for CFS [2] that includes the following criteria: fatigue lasting longer than 6 months or more, no other illness that could explain the fatigue, 4–8 concurrent symptoms, fatigue that is not relieved by rest, and fatigue that interferes with occupational, educational and social or personal activities.

Healthy controls were recruited through advertisements. They were similar in age, sex, and activity level (sedentary to moderately active) to the CFS patients. All women, were scheduled for exercise challenge 5 to 10 days after the first day of their menstrual cycle. All subjects were asked not to use inhaled or systemic corticosteroids, anti-histamines, or anti-inflammatory medication for 7 days prior to the exercise challenge. Subjects performed a submaximal (70% predicted maximum work load), steady-state exercise for 20 minutes on a stationary bicycle ergometer. All subjects met these challenge criteria. Blood samples were obtained immediately before and 24 hours following exercise. The selection of subjects for inclusion into the gene expression pilot study, focused on those without allergies, to whom an exercise challenge was given: 5 women with CFS and 5 female controls.

### RNA isolation

Immediately following blood collection, peripheral blood mononuclear cells were isolated, using Ficoll gradients and stored in RNAqueous™ lysis buffer (Ambion Inc., Austin, TX) at -70°C. Total RNA was extracted using the RNAqueous™ kit (Ambion), with quality and quantity assessed by agarose gel electrophoresis as described previously [23].

### Preparation and hybridization of labeled cDNA

Biotinylated cDNA synthesis from 1 μg of total RNA, microarray hybridization, and detection were performed as previously described [23]. Atlas™ Human 3.8I oligonucleotide glass microarrays (Clontech Laboratories, Palo Alto, CA) were used. The slides were archived and images captured using the GSD-501™ scanner (Invitrogen Life Technologies, Carlsbad, CA).

### Data analysis

#### Preprocessing data

The scanned TIFF images were processed using ArrayVision™ (Imaging Research Inc., Ontario, Canada) as previously described [23]. A median background value was calculated around each of the 3757 features and subtracted from the mean feature signal to give the net signal for the respective gene. Values were log_2_-transformed, and a global normalization was used to median center the log intensity values. Genes whose expression differed by at least 1.5 fold from the median in at least 20% of arrays were retained (3699 genes), thus excluding genes showing minimal variation across the set of arrays. If an expression value was missing or filtered out in more than 50% of the arrays, that feature was not included, leaving a total of 3682 genes for analysis.

#### Analysis

We used several analytical approaches to look for differential expression among four predefined classes: control subjects before exercise, control subjects after exercise, CFS subjects before exercise, and CFS subjects after exercise. Comparisons included paired analyses of controls before versus after exercise and CFS cases before versus after exercise. In addition, we examined pre-exercise (baseline) controls versus pre-exercise CFS subjects and post-exercise controls versus post-exercise CFS subjects.

##### Analytical approach 1

The more commonly used approach to microarray gene expression analysis is to establish if gene expression profiles differ between subjects in predetermined classes and to identify the genes responsible for the differences. The Class Comparison analysis method in BRB ArrayTools [24] uses this approach, applying a random variance t-test to the data. This is an improvement over the standard t-test as it permits sharing information among genes about within-class variation without assuming that all genes have the same variance [25]. It is an advisable modification when the numbers of samples per class are small. Genes were considered statistically significant if the parametric p-value was less than 0.005. Next a global, multivariate permutation test was performed to determine whether the expression profiles differed between the classes by permuting the array classification. We used this test to provide a median false discovery rate of 10%.

The EASE software package [26] was then used to evaluate the biologic significance of the ontology of genes identified as differentially expressed by class comparison. EASE performs a statistical analysis of gene categories in a gene list relative to all genes on the array and calculates a conservative variant of the standard Fisher exact probability called the EASE score. The most significant categories as assessed by EASE score are deemed "themes" of the gene list. These themes correspond to the systematic and standardized nomenclature developed by the GO (Gene Ontology) Consortium [27]. The three organizing principles of GO are molecular function, biological process and cellular component, and presently 22,665 human gene products have been annotated. Associating genes with related GO terms assists in the interpretation of expression patterns. A GO category includes genes described by that term and those included in any subset (or children) of that GO term. A gene may be categorized in more than one ontology category.

To demonstrate the distinct gene clusters, we performed a two-way hierarchical cluster analysis on the genes identified by this analytical approach, using the algorithm described by Eisen et al. [28].

##### Analytical approach 2

We used the Gene Ontology comparison module of BRB Array tools [24] as a second analytical approach. In this method, the genes are assigned GO terms prior to analysis. For each GO term the total number of genes on the array belonging to that category was determined. A random variance t-test or F-test [25] was used to determine the p-value for differences between the predefined classes for each gene in the GO term. Two statistics are computed to summarize the p-values for all genes in the GO term: the Fisher statistic and the Kolmogorov-Smirnov statistic [24]. These p-values provide a list of GO categories that have more genes differentially expressed among the classes than expected by chance. We considered a GO category differentially regulated if either significance level was less than 0.005. All GO categories with between 5 and 100 genes represented on the array were considered.

## Competing interests

The author(s) declare that they have no competing interests.

## Authors' contributions

TW contributed to the design of the experimental approach, the analysis of the data and drafted the manuscript. JFJ contributed to the conception and design of this study, conducted the exercise challenges and assisted in drafting the manuscript. ERU contributed to the design of the experimental approach, and to the manuscript preparation. SDV was instrumental in the design of the experimental approach, analysis, presentation, discussion of these data and manuscript preparation. All authors read and approved the final manuscript.

## Figures and Tables

**Table 1:** Demographic and challenge related information of subjects included in this study.

**Exercise Challenge Information**	**Subjects**	
	
	**CFS**	**Controls**
Mean Age	41.8	36.1
Age Range	35–48	27–45
Average workload performed during exercise challenge in watts.	1360	1664
Mean change in score of daily symptom diary (± SE) calculated for the exercise challenge.^#^	1.61 ± 0.65*	0.54 ± 0.31

^#^A 10 symptom daily diary with a 0 to 4 scale was filled out each day for 2 weeks before and 1 week after challenge [22].

*Significant relative to control patients at P < 0.05

**Table 2:** List of genes differentially expressed in exercised control subjects. The parametric p-value is a measure of the significance of the random variance t-statistic test used to identify differentially expressed genes in the class comparisons.

**GenBank ID**	**Description**	**Parametric p-value**
NM_003431	Zinc finger protein 124 (HZF-16)	0.0001
NM_003466	Paired box gene 8	0.0001
NM_003477	Pyruvate dehydrogenase complex, lipoyl-containing component X; E3-binding protein	0.0002
NM_003591	Cullin 2	0.0003
NM_003985	Tyrosine kinase, non-receptor, 1	0.0004
NM_003490	Synapsin III	0.0004
NM_003764	Syntaxin 11	0.0004
NM_003494	Dysferlin, limb girdle muscular dystrophy 2B (autosomal recessive)	0.0006
NM_003715	Vesicle docking protein p115	0.0006
NM_003692	Transmembrane protein with EGF-like and two follistatin-like domains 1	0.0006
NM_003853	Interleukin 18 receptor accessory protein	0.0006
NM_003716	Ca^2+^-dependent activator protein for secretion	0.0006
NM_003558	Phosphatidylinositol-4-phosphate 5-kinase, I beta	0.0008
NM_003854	Interleukin 1 receptor-like 2	0.0009
NM_003543	H4 histone family, member H	0.0010
NM_003488	A kinase (PRKA) anchor protein 1	0.0013
NM_003487	TATA box binding protein (TBP)-associated factor, RNA polymerase II, N, 68 kD	0.0014
NM_003528	H2B histone family, member Q	0.0019
NM_003693	Acetyl LDL receptor; scavenger receptor expressed by endothelial cells	0.0021
NM_003473	Signal transducing adaptor molecule (SH3 domain and ITAM motif) 1	0.0029
NM_004653	SMC (mouse) homolog, Y chromosome	0.0035
